# Interobserver reproducibility of perineural invasion of prostatic adenocarcinoma in needle biopsies

**DOI:** 10.1007/s00428-021-03039-z

**Published:** 2021-02-03

**Authors:** Lars Egevad, Brett Delahunt, Hemamali Samaratunga, Toyonori Tsuzuki, Henrik Olsson, Peter Ström, Cecilia Lindskog, Tomi Häkkinen, Kimmo Kartasalo, Martin Eklund, Pekka Ruusuvuori

**Affiliations:** 1 Department of Oncology and Pathology, Karolinska Institutet, Karolinska University Hospital, Radiumhemmet P1:02, 171 76 Stockholm, Sweden; 2 Department of Pathology and Molecular Medicine, Wellington School of Medicine and Health Sciences, University of Otago, Wellington, New Zealand; 3 Aquesta Uropathology and University of Queensland, Brisbane, Queensland Australia; 4 Department of Surgical Pathology, Aichi Medical University, School of Medicine, Nagoya, Japan; 5 Department of Medical Epidemiology and Biostatistics, Karolinska Institutet, Stockholm, Sweden; 6 Department of Immunology, Genetics, and Pathology, Uppsala University, Uppsala, Sweden; 7 Faculty of Medicine and Health Technology, Tampere University, Tampere, Finland; 8 Tays Cancer Center, Tampere University Hospital, Tampere, Finland; 9 Institute of Biomedicine, University of Turku, Turku, Finland

**Keywords:** Pathology, Reproducibility, Perineural invasion, Prostate cancer

## Abstract

Numerous studies have shown a correlation between perineural invasion (PNI) in prostate biopsies and outcome. The reporting of PNI varies widely in the literature. While the interobserver variability of prostate cancer grading has been studied extensively, less is known regarding the reproducibility of PNI. A total of 212 biopsy cores from a population-based screening trial were included in this study (106 with and 106 without PNI according to the original pathology reports). The glass slides were scanned and circulated among four pathologists with a special interest in urological pathology for assessment of PNI. Discordant cases were stained by immunohistochemistry for S-100 protein. PNI was diagnosed by all four observers in 34.0% of cases, while 41.5% were considered to be negative for PNI. In 24.5% of cases, there was a disagreement between the observers. The kappa for interobserver variability was 0.67–0.75 (mean 0.73). The observations from one participant were compared with data from the original reports, and a kappa for intraobserver variability of 0.87 was achieved. Based on immunohistochemical findings among discordant cases, 88.6% had PNI while 11.4% did not. The most common diagnostic pitfall was the presence of bundles of stroma or smooth muscle. It was noted in a few cases that collagenous micronodules could be mistaken for a nerve. The distance between cancer and nerve was another cause of disagreement. Although the results suggest that the reproducibility of PNI may be greater than that of prostate cancer grading, there is still a need for improvement and standardization.

## Background

Perineural invasion (PNI) is a well-known route of dissemination of prostatic adenocarcinoma. The prognostic value of PNI for prostate cancer has been confirmed in recent meta-analyses [1, 2]. Numerous studies have found a correlation of PNI in core needle biopsies with stage [1, 3, 4] and with outcome after radical prostatectomy [1, 2, 4] or radiotherapy [2, 5, 6]. Whether the presence of PNI in needle biopsies is an independent predictor of outcome, when other histopathological characteristics are factored in, is controversial [1, 7]. The reporting of PNI in needle biopsies is thus a recommended, but not a required element, in the International Collaboration on Cancer Reporting (ICCR) dataset [8].

The practical utility of histopathologic-derived prognostic factors is hampered by interobserver variability. Numerous reports have been published on the reproducibility of prostate cancer grade [9–12], but few studies have been undertaken on the reproducibility of stage-related parameters [13]. In particular, little is known regarding the intra- and interobserver variability of diagnosing PNI in needle biopsies.

The aim of this study was to analyze the reproducibility of the identification of PNI in a series of prostate biopsies from a population-based cohort and also to identify possible diagnostic pitfalls.

## Materials and methods

The Stockholm-3 study was a population-based prostate cancer screening study undertaken among men aged 50–69 years during the years 2012–2015 [14]. Core needle biopsies from a total of 7406 men were reviewed by a single pathologist (L.E.). As PNI is more commonly found in high-grade prostate cancer and a majority of cancers detected by screening is low grade, the series was enriched by PNI cases from high-grade cancers. Biopsies of 1427 men were selected for digital scanning, including biopsies from all patients positive for PNI (*n*=266) supplemented with a random selection stratified by Gleason score (*n*=1161) to enrich the series for high-grade cases. From this series, 106 biopsy cores with PNI and 106 cores without PNI were selected. The distribution of biopsies with PNI was blinded for all observers.

The glass slides were scanned at ×20 lens magnification, and digital slides were circulated among four experienced pathologists with a special interest in urological pathology (B.D., L.E., H.S., T.T.), for assessment of PNI using Cytomine, a software for image-based collaborative studies [15]. Features accepted as PNI by all authors included cancer surrounding a nerve (circumferential PNI), cancer partly surrounding or abutting on a nerve, and cancer infiltrating a nerve. In cases where there was uncertainty as to the diagnosis, the participants were asked to add a comment that it was a borderline case and specify if the problem was uncertainty as to whether the structure adjacent to cancer was a nerve or if the distance from cancer to nerve was small enough to justify a diagnosis of PNI. There was also an option to indicate Borderline Other if there was, for example, uncertainty as to whether the epithelial structure next to a nerve was malignant or benign. The time taken to evaluate each core by each of the observers was automatically registered.

In a second round, 52 cases where there were discrepancies between at least two observers were re-circulated. The observers encircled areas where they had found PNI in the first round. The discrepant cases were then investigated by immunohistochemistry for S-100. Paraffin sections were cut at 4 μm, deparaffinized in xylene, and rehydrated through graded ethanol. For antigen retrieval, the slides were treated in Diva Decloaker ×20 (BioCare Medical, Pacheco, California, USA) for 40 min at 95 C. Sections were incubated 30 min with mouse monoclonal antibodies against S-100 protein (ab4066, Abcam, Cambridge, UK), at 1:100 dilution. For biotin-free detection of primary antibodies, a MACH 1 HRP-polymer detection kit was used and then visualized by beta DAB (BioCare Medical, Pacheco, California, USA). The slides were counterstained with Mayer’s hematoxylin. For positive controls, selected needle biopsies with obvious peripheral nerves were used, and for negative controls, slides were incubated without primary antibody. The immunostained slides were reviewed by one observer (L.E.) and compared with the digitized sections that were used for the interobserver reproducibility study. It was assessed as to whether or not the observed structures that were suggestive of PNI on hematoxylin and eosin–stained sections were present in the immunostains and if immunohistochemistry verified PNI.

Mean pairwise Cohen’s kappa for each of the pathologists against the other observers was computed in accordance with previous publications [10]. Sensitivity, specificity, and positive and negative predictive values and accuracy were calculated, based on the results of cases where there was either a 100% agreement among the observers or was determined by immunostaining that confirmed the presence or absence of PNI.

## Results

Cancer was diagnosed in 2810 (37.8%) of the Stockholm-3 biopsies. PNI was reported in 579 (20.7%) of all cancers and was more commonly seen in high-grade tumors (Table 1). The grade distribution of the 212 selected cores of the current study and the rate of PNI by Gleason scores according to original reports are given in Table 2.

**Table 1:** Gleason score distribution of cancer biopsies in the STHLM3 study and distribution of perineural invasion by grade

Gleason scores	Number of cancers (%)	Number of PNI cases (%)
6	1558 (55.6%)	113 (7.3%)
7 (3+4)	761 (27.2%)	162 (21.3%)
7 (4+3)	253 (9.0%)	102 (40.3%)
8	101 (3.6%)	94 (93.1%)
9–10	128 (4.6%)	108 (84.4%)
Total	2801 (100%)	579 (20.7%)

**Table 2:** Gleason score distribution of biopsies in the current study

Gleason scores	All biopsies (%)	Biopsies with PNI (%)	Biopsies without PNI (%)
6	80 (37.7%)	17 (16.0%)	63 (59.4%)
7 (3+4)	39 (18.4%)	21 (19.8%)	18 (17.0%)
7 (4+3)	29 (13.7%)	22 (20.8%)	7 (6.6%)
8	33 (15.6%)	22 (20.8%)	11 (10.4%)
9–10	31 (14.6%)	24 (22.6%)	7 (6.6%)
Total	212 (100%)	106 (100%)	106 (100%)

PNI was diagnosed by all four observers in 34.0% (72/212) of cases while 41.5% (88/212) were considered by all to be negative for PNI. In 24.5% (52/212) of cases, there was a disagreement, with PNI being reported by 1, 2, or 3 observers in 14, 14, and 24 cases, respectively. The kappa for interobserver variability was 0.67–0.75 (mean 0.73) for the four observers (Table 3). The median time for reviewing one case varied from 39 to 116 s among the observers. The median time for all cases was 81 s (Table 3).

**Table 3:** Results of individual observers. Mean pairwise Kappa (95% confidence intervals)

Observer	Kappa	Cores with PNI (*n*)	Median time per case (s)
1	0.75 (0.68–0.82)	114	113
2	0.75 (0.69–0.82)	103	79
3	0.75 (0.69–0.82)	106	116
4	0.67 (0.59–0.76)	79	39
	Mean 0.73	Mean 106	Median 81

One of the observers (L.E.) had reported the glass slides of the biopsies 5 to 7 years earlier, and for this study, reviewed scanned slides blinded as to the results of the original reports. The kappa for intraobserver variability was 0.87, when comparing results from glass slides and scanned slides. In 93.4% (198/212) of biopsies, the diagnosis was unchanged, while in 5.2% (11/212), the previous diagnosis was changed from no PNI to PNI, and in 1.4% (3/212), the diagnosis was changed from PNI to no PNI.

A borderline diagnosis was reported by any of the four observers in 29.7% (63/212) of biopsies. In 54 of these, a borderline diagnosis was assigned by only one observer. The total number of borderline diagnoses given by any observer was 77, and of these, the most common comment was borderline nerve (50), followed by borderline distance (21) and borderline other (6). The borderline labels were almost equally distributed in cases with a diagnosis of PNI (39) and no PNI (38). Each observer reported 14, 17, 22, and 24 borderline cases, respectively. Of the 63 cases with at least one borderline diagnosis, PNI was reported by all observers in 13 cases and by none in 16 cases, and in 34 cases, the observers were discordant.

Among the 52 biopsies, 49 had paraffin blocks available for immunohistochemistry. In 14 biopsies, the observed structures had been lost at re-cut. The immunostains of the remaining 35 included the structures that were suspicious for PNI on hematoxylin and eosin staining. Of these, 31 had PNI, while 4 had no PNI. In 195 cases, there was either a 100% agreement among the observers for or against PNI (72 and 88, respectively) or an available immunostain that confirmed the diagnosis (35). Among the 195 biopsies, 52.8% had PNI (103/195) while in 47.2% (92/195) of biopsies, there was no PNI. Based on these cases, the sensitivity, specificity, and positive and negative predictive values and accuracy for PNI of the observers were calculated (Table 4).

**Table 4:** Sensitivity, specificity, positive and negative predictive values (PPV and NPV) and accuracy (%)

Observer	Sensitivity	Specificity	PPV	NPV	Accuracy
1	98.1	98.9	99.0	97.8	98.5
2	87.4	97.8	97.8	87.4	92.3
3	90.3	98.9	98.9	90.1	94.4
4	75.7	100.0	100.0	78.6	87.2
Mean	87.9	98.9	98.9	88.5	93.1

**Fig. 1:**
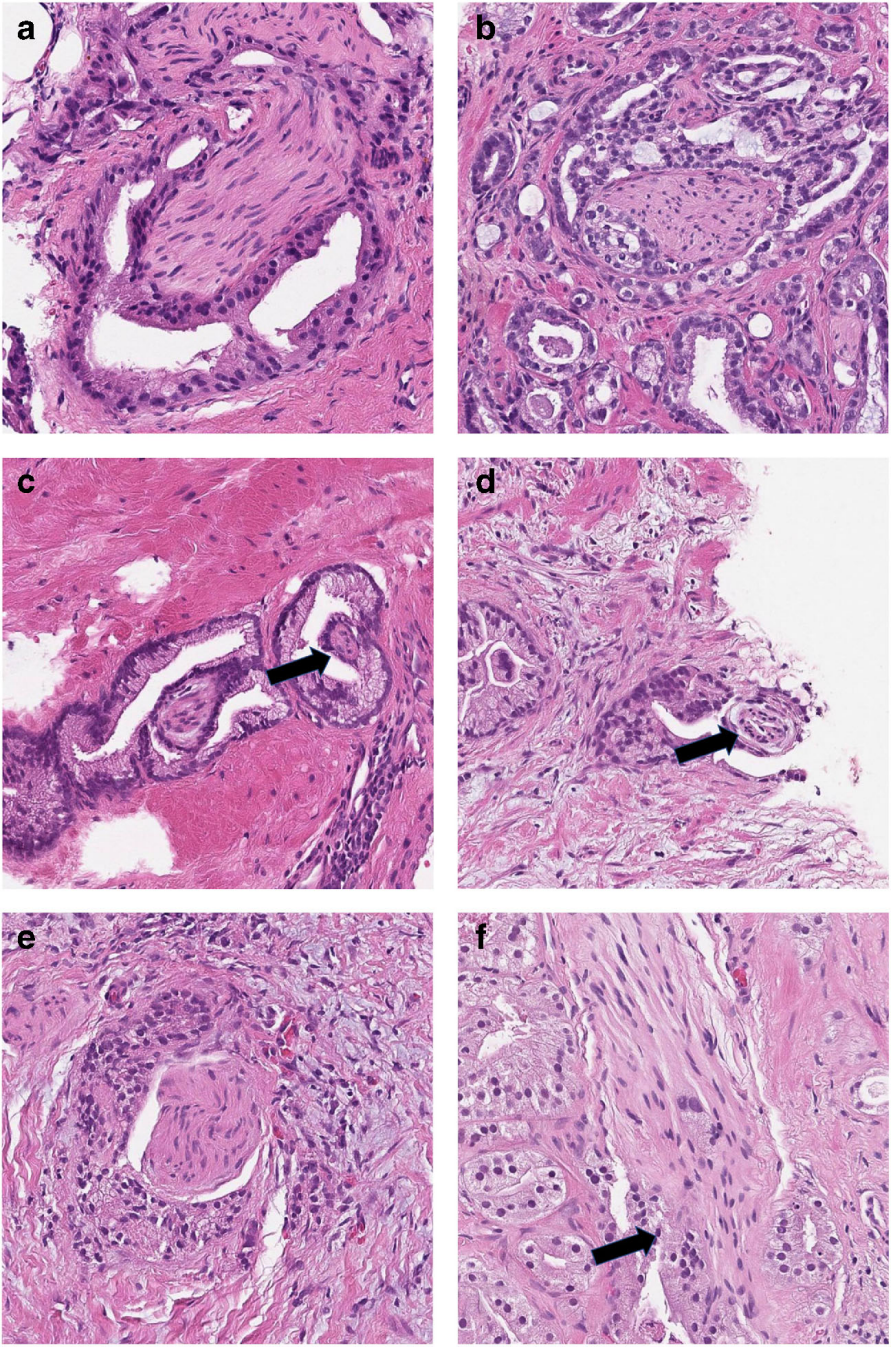
**a**–**f** Cases with agreement for PNI among all four observers. **a** Longitudinal section through nerve with fibrillary material and thin nuclei with wavy shape and tapering ends. **b** Cross-section through nerve. Fibrillary structure is still evident, but the nuclei are mostly rounded when cut across. **c** Two nerves with PNI. One of the nerves mimic fibrous stroma of papillary infolding. Such structures are not uncommonly nerves. Here, the diagnosis is evident by the resemblance with the more obvious nerve to the left. **d** Small nerve that has been cut across. Basophilic mucinous material in perineural space helps recognizing it as a nerve. **e** A retraction cleft between nerve and cancer is sometimes seen in PNI. **f** Longitudinal section through nerve with cancer impinging upon the nerve

**Fig. 2:**
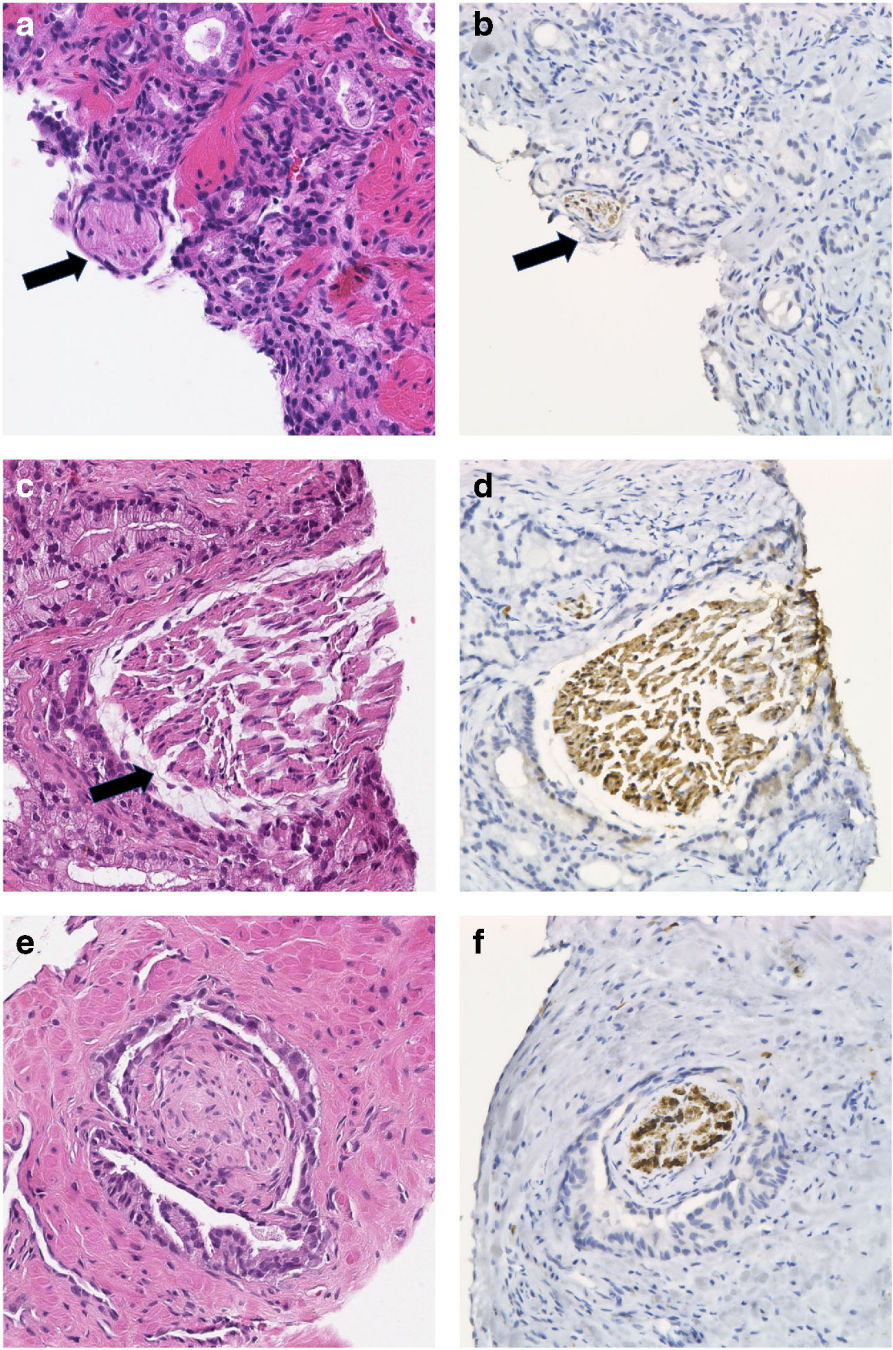
**a**–**f** Cases with disagreement for PNI among the observers, but confirmed by immunohistochemistry for S-100. **a**, **b** Minimal nerve-like structure that may be difficult to diagnose because of its small size (arrows), here confirmed by immunohistochemistry. **c**, **d** Cross-section through a large nerve that has some resemblance with a smooth muscle bundle. The distance between the nerve and the surrounding cancer (arrow) also caused diagnostic concern. **e**, **f** This case caused uncertainty both as to whether the surrounded structure was a nerve or a smooth muscle bundle and whether the surrounding cancer was close enough to the nerve to justify cancer. Circumferential PNI favors cancer under the condition that the central structure really is a nerve. It was also reported as Borderline Other as it was uncertain if the surrounding gland with papillary folds and minimal atypia was malignant. Immunohistochemistry for S-100 confirmed the presence of a nerve, and negative staining for p63 (not shown) confirmed that the gland was cancerous. **a**, **c**, **e** Hematoxylin and eosin. **b**, **d**, **f** Immunohistochemistry for S-100

**Fig 3:**
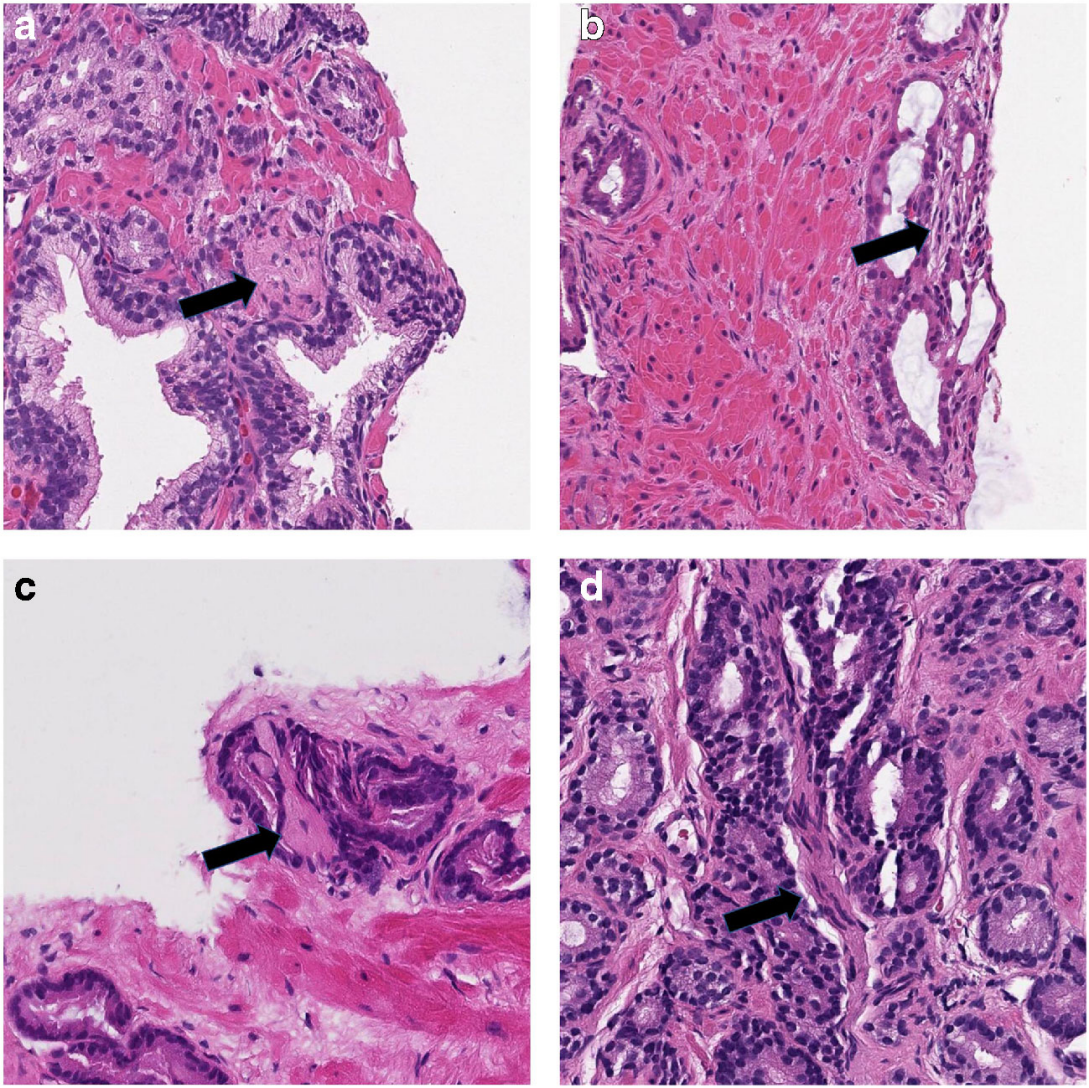
**a**–**d** Cases with disagreement for PNI among the observers, but negative S-100 stain refuting a diagnosis of PNI (not shown). **a**, **b** Stromal structures with some fibrillary structure and thin, dark nuclei, but not enough distinct for a definitive diagnosis of PNI. **c** Pale-staining, almost acellular structure with some fibrillary material adjacent to cancer, most likely small collagenous micronodules. **d** Eosinophilic bundle of cells with slender dark nuclei. Negative S-100 stain favors reactive stroma or smooth muscle bundle

Examples of PNI with complete agreement among the observers are shown in Fig. 1a–f. Figure 2 a–f show the cases where the diagnosis of PNI was contentious but verified by immunohistochemistry. In Fig. 3a–d, examples are given of differential diagnoses that were found to be negative for PNI by immunohistochemistry.

In the 35 discordant cases, where immunohistochemical stains could be matched against the observed morphological findings, one or several of the following features accounted for the diagnostic difficulties. The most common differential diagnosis to PNI was bundles of stroma or smooth muscle tissue (Fig. 3a, b), which was observed in 18 cases. In eight cases, obvious neural structures were overlooked by at least one observer. In two cases, there was a resemblance to collagenous micronodules (Fig. 3c), while in six cases, the structures suspicious for PNI were minimal and difficult to classify on hematoxylin and eosin–stained sections (Fig. 2a and 3a, b). The distance of the tumor to the nerve caused a diagnostic problem for at least one observer in seven of cases, which was stated by the comment of borderline distance (Fig. 2c–f).

## Discussion

The predictive and prognostic role of PNI of prostate carcinoma in needle biopsies has been debated at length. Numerous studies have indicated a correlation of PNI in biopsies with stage [3, 4] and with progression after radical prostatectomy [1, 4, 16] or radiotherapy [2, 5, 6]. In a meta-analysis of the prognostic impact of PNI, the majority of studies showed an increased risk of biochemical recurrence after radical prostatectomy or radiotherapy [2]. Uncertainty remains as to whether PNI is an independent predictor of outcome when other available prognostic indicators are taken into account, although in several studies, PNI in needle biopsies predicted death of disease after radical prostatectomy in multivariable analysis [4, 16]. Similarly, PNI independently predicted bone metastases after radiotherapy in a series of men with locally advanced prostate cancer [5]. However, in a recent report on an active surveillance cohort, biopsy PNI was not an independent predictor of death due to prostate cancer when grade, serum PSA, tumor extent, and clinical stage were included in a multivariable analysis [7]. In another recent study, PNI in biopsies did not predict upgrading at radical prostatectomy [17]. Thus, it seems that the utility of reporting PNI in needle biopsies depends on the treatment given and the chosen clinical endpoint.

The reported rate of PNI in needle biopsies varies widely. Some of the variation depends on patient selection. For example, the PNI incidence was 20.7% in a screening trial where the majority of patients were diagnosed with low-grade cancer [14] (Table 1) and 46% in a radiotherapy trial, which included men with locally advanced cancer [5]. However, it is apparent that the reported incidence of PNI also depends on how data were collected. In studies on localized prostate cancer that were either prospective or retrospective, with centralized biopsy review, PNI was typically reported in 20–29.1% of cases [4, 6, 7, 16], while in some retrospective studies, based on original pathology reports, the PNI rate has been as low as 6.7–7% [18, 19]. Another reason behind this wide variation is that the diagnosis of PNI is subjective, a feature that it has in common with other histopathological parameters. Few studies have addressed the reproducibility of PNI, and little work has been done on standardizing criteria for its diagnosis.

In this study, we assessed the interobserver reproducibility of PNI among experts in uropathology and analyzed causes of disagreement. The four observers achieved a mean kappa of 0.73 (0.67–0.75). This is a relatively high level of concordance when compared with grading studies, where weighted kappa for agreement among expert pathologists has usually been in the range of 0.5–0.7 [10, 11, 20]. A possible explanation for these more favorable results is that the diagnosis of PNI may be easier to achieve than the compilation of more complex and heterogeneous-grade information. To our knowledge, interobserver reproducibility of PNI has only been reported in one previous study, where four pathologists assessed several parameters on 50 needle biopsies, including PNI, with the aim of comparing the results of digital and routine microscopic assessment without immunohistochemical verification [21]. The interobserver kappa values for PNI in that study were 0.55 and 0.65 on routine and digital microscopic examination, respectively, i.e., slightly lower than in the current study. The reproducibility of other stage-related parameters was investigated by Evans et al. [13]. A group of 12 urological pathology experts assessed scanned slides from 60 radical prostatectomy specimens and achieved an overall kappa value of 0.74 for surgical margin status and 0.63 for extraprostatic extension.

We also evaluated the intraobserver reproducibility of PNI, and a kappa value of 0.87 was achieved, which is within the range usually classified as almost perfect. The washout period in the study was at least 5 years which ensured an unbiased assessment. It is not surprising that some intraobserver disagreement occurred as the comparison was undertaken between scanned slides and glass slides, which have different optical resolution. However, digital microscopy has been used in several previous studies with satisfactory results [22, 23]. Since the focus of the study was the identification of PNI, the observer may have made a greater effort in searching for PNI in the study slide review than had been achieved in the original reporting, as shown by the detection of PNI in an additional 5.4% of cases. Although the initial reporting was undertaken at core level, the effort of identifying PNI in individual cores may have been limited. This accords with the ICCR recommendations that it is sufficient to report PNI in the summary diagnosis of biopsies and that core level reporting of PNI is not considered necessary [8]. By comparison, Rodriguez-Urrego et al. reported a slightly lower intraobserver reproducibility, with a kappa of 0.71 (0.53–0.84) [21].

Peripheral nerves in histological sections of prostate tissue have different appearances depending on their size and how they are cut. The classical slender wavy nuclei with tapering ends are seen when nerves have been cut longitudinally, while transverse cutting produces a rounded bundle with round nuclei that may resemble a bundle of smooth muscle. Several differential diagnoses of PNI were reported in the study, including bundles of smooth muscle or fibrous tissue (Fig. 3a, b) and collagenous micronodules (Fig. 3c). Another problem is that pathologists may suspect PNI but feel uncertain as to whether a nerve-like structure is definitely a nerve. This is reflected in the finding that 29.7% of biopsies were considered borderline PNI by at least one of the observers.

The distance between cancer and a nerve was a source of hesitation in 8% of cases. The definition of PNI is not self-evident. In 1985, Batsakis defined PNI as “tumor cell invasion in, around, and through the nerves” [24]. Others have suggested “tumor cells within any of the 3 layers of the nerve sheath or tumor foci outside of the nerve with involvement of ≥33% of the nerve’s circumference” [25]. This latter definition would thus not require the presence of cancer growing in the perineural space. In cases of PNI, there may be a small retraction space between the nerve and cancer (Fig. 1e). The acceptable width of this space to negate a diagnosis of PNI is at present a judgment call and hence a source of interobserver variability.

The time spent on reviewing the slides varied widely between the observers. The slowest observer spent 116 s per case to search for PNI, which would translate to 23 min for a 12-core set of biopsies. This is slower than normal speed for the reading of prostate biopsies and may be explained by the time-consuming procedure of reading digitized slides. The two slowest observers took almost three times as long to review the cases as the fastest. Interestingly, the two slowest observers also detected 34% and 44% more cores with PNI, suggesting that time spent examining tissue is important for the detection of PNI.

Considerable effort has been committed to the standardization of grading of prostate cancer. The few studies to date that have focused on stage-related parameters for prostate cancer indicate that there is still a need for improvement in both the standardization of parameters and their reproducible identification. There is accumulating evidence that PNI on needle biopsies has prognostic value, and by implication, this increases the importance of providing accurate reporting of PNI.

## Declarations

The study was approved by the Regional Ethic Review Board, Stockholm (2012/572-31/1, 2018/845-32).
